# The ADAR RNA editing enzyme controls neuronal excitability in *Drosophila melanogaster*

**DOI:** 10.1093/nar/gkt909

**Published:** 2013-10-16

**Authors:** Xianghua Li, Ian M. Overton, Richard A. Baines, Liam P. Keegan, Mary A. O’Connell

**Affiliations:** ^1^MRC Human Genetics Unit, Institute of Genetics and Molecular Medicine at the University of Edinburgh, Crewe Road, Edinburgh EH4 2XU, Scotland, UK, ^2^Faculty of Life Sciences, University of Manchester, Oxford Road, Manchester M13 9PT, UK and ^3^Department of Molecular Biosciences, The Wenner Gren Institute, Stockholm University, Svante Arrhenius väg 20C, 106 91 Stockholm, Sweden

## Abstract

RNA editing by deamination of specific adenosine bases to inosines during pre-mRNA processing generates edited isoforms of proteins. Recoding RNA editing is more widespread in *Drosophila* than in vertebrates. Editing levels rise strongly at metamorphosis, and *Adar^5G1^* null mutant flies lack editing events in hundreds of CNS transcripts; mutant flies have reduced viability, severely defective locomotion and age-dependent neurodegeneration. On the other hand, overexpressing an adult dADAR isoform with high enzymatic activity ubiquitously during larval and pupal stages is lethal. Advantage was taken of this to screen for genetic modifiers; *Adar* overexpression lethality is rescued by reduced dosage of the *Rdl (Resistant to dieldrin),* gene encoding a subunit of inhibitory GABA receptors. Reduced dosage of the *Gad1* gene encoding the GABA synthetase also rescues *Adar* overexpression lethality. *Drosophila Adar^5G1^* mutant phenotypes are ameliorated by feeding GABA modulators. We demonstrate that neuronal excitability is linked to dADAR expression levels in individual neurons; *Adar-*overexpressing larval motor neurons show reduced excitability whereas *Adar^5G1^* null mutant or targeted *Adar* knockdown motor neurons exhibit increased excitability. GABA inhibitory signalling is impaired in human epileptic and autistic conditions, and vertebrate ADARs may have a relevant evolutionarily conserved control over neuronal excitability.

## INTRODUCTION

RNA editing by ADARs (adenosine deaminases acting on RNA) has been proposed to diversify transcripts, particularly in the brain, to meet the physiological needs of the organism (1,2). The ADAR enzymes convert specific adenosines to inosines within duplex regions of transcripts. Many of the transcripts edited by ADARs are expressed in the central nervous system (CNS). Ribosomes decode inosine as guanosine, and editing events within open reading frames lead to production of edited isoforms of ion channel subunits and other proteins. Production of edited subunit isoforms affects the pharmacological properties of channels and receptors such as AMPA receptors, potassium Kv1.1 channels and serotonin 5HT_2c_ receptors (3–6). Various neurological disorders including Motor Neuron Disease, depression and epilepsy have been linked to defects in RNA editing, particularly to ADAR2 and to the transcripts that it edits (7–11). Developmental and tissue-specific ADAR regulation is also important, and both edited and unedited isoforms of channels have function; unedited GABA_A_ (γ-aminobutyric acid) receptor, for instance, is crucial for synapse formation in the developing vertebrate brain (12,13).

Homozygous *Adar2* null mice die at or before weaning owing to seizures principally attributed to loss of editing at the *Q/R* site in the *Gria2* (previously *GluR-2*), transcript encoding subunit 2 of the glutamate receptor, resulting in the production of abnormal calcium-permeable AMPA receptors (14,15). The *Gria2 Q/R* site is constitutively edited to 100% efficiency (16–18). Editing at this site is crucial for viability, and mutating the chromosomal *Gria2* gene to express only the GRIA2 R edited isoform rescues the *Adar2* mutant lethality (14). Prevention of excessive Ca^2+^ influx through GRIA 2*-*containing AMPA receptors appears to be the primary function of ADAR2 in mice (15,16,19).

*Drosophila Adar* is a homologue of mammalian *Adar2* (20), although *Drosophila* transcripts encoding subunits of excitatory glutamate receptors are not edited (21). Despite the loss of editing at specific sites in a large set of transcripts, *Drosophila Adar* null mutant flies are viable and morphologically normal. However *Adar* mutant flies do show uncoordinated locomotion, loss of the male courtship display, leg tremors, temperature-sensitive paralysis and age-dependent progressive neural degeneration (21,22). The defects of the *Adar* mutant are likely to derive from malfunctioning of multiple membrane channels and trafficking proteins that comprise the largest functional grouping among the edited transcripts (21,23,24). Although effects of editing on several different *Drosophila* ion channels have been characterized after *Xenopus* oocyte expression, the overall physiological role of ADAR-mediated RNA editing is incompletely understood in either insects or mammals (15,25–27). Recently, *Drosophila* ADAR was shown to limit synaptic release at larval neuromuscular junctions (NMJs) (28), raising the possibility that the major physiological role of ADAR is in modulating neuronal activity and/or maintaining neural homeostasis.

Expression of *Adar* transcripts from the chromosomal gene is low at the earlier stages of development and increases at metamorphosis. We also previously described a potential autoregulatory circuit in adult flies in which ADAR proteins edit the *Adar* transcript to produce an ADAR isoform with lower RNA editing activity (29). In adult flies, which express primarily the more active *Adar 3/4* splice form, dADAR protein edits Serine (S) codon 391 to Glycine (G), changing a residue on the RNA-binding face of the deaminase domain. The ‘genome-encoded’ dADAR 3/4S protein has an 8-fold higher editing efficiency *in vitro* than the edited dADAR 3/4 G isoform after purification from overexpressing yeast cultures (29).

An ‘ineditable’ *UAS-Adar 3/4 S* cDNA construct in which the self-editing (S/G) site is mutated to an alternate serine codon ‘TCT’, which cannot be edited, produces only the dADAR 3/4S isoform (29). Overexpression of *Adar 3/4S* in *Drosophila* under the control of the *Actin 5c-GAL4* driver, which expresses ubiquitously and strongly from embryonic stages, is lethal in larvae and pupae. This is probably because the overexpressed *Adar 3/4S* bypasses the normal self-limitation of dADAR RNA editing activity. Lethality depends on the editing activity of dADAR 3/4S. Taking advantage of the power of *Drosophila* genetics, we performed a screen for genetic suppressors of this *Adar* overexpression lethality. We find that genetic manipulation to reduce GABA signalling rescues the lethality caused by dADAR 3/4S overexpression. We present evidence that *Drosophila* ADAR acts cell-autonomously to fine-tune neural activity. *In vivo* extracellular recordings show that dADAR 3/4S overexpression dampens larval motor neuron excitability. Also, *Adar^5G1^* null flies exhibit increased neuronal excitability and viability, and locomotion defects are rescued by chemical enhancement of GABA signalling. Our data indicate that dADAR is required to protect neurons from hyper-excitability and that dADAR suppresses neuronal excitability in concert with GABA signalling.

## MATERIALS AND METHODS

### *Drosophila* strains

Fly stocks were maintained at 18°C on a 12 h light/dark cycle and were raised on standard corn meal agar media.

***w^1118^*:** Wild-type strain. ***Adar 3/4S OE*:** *w^1118^; Actin 5c-GAL4 (25FO1) /SM5 CyO; UAS-Adar 3/4S (14), Tub-GAL80^ts^(2), /UAS-Adar 3/4S (14), Tub-GAL80^ts^(2) (or TM3 Sb)* has a homozygous lethal *Actin 5c-GAL4* insert and a *Tub-GAL80^ts^* insert combined with *UAS-Adar 3/4S*. ***Adar^5G1^/FM7*a:** *y, Adar^5G1^, w/FM7a. Adar* null with *FM7a Bar* balancer. ***Adar^5G1^/FM7,GFP*** *y, Adar^5G1^, w/FM7c, P{GAL4-Kr.C}DC1, P{UAS-GFP.S65T}DC5* is *Adar* null with *FM7,GFP* balancer. ***RRa-GFP*:** *UAS-mCD8-GFP; RRA-GAL4* has the larval aCC motor neuron GAL4 driver with GFP expression. ***Adar siRNA***: Transformant ID7763 (VDRC). ***Rdl siRNA***: Transformant ID 41103 (VDRC), Transformant ID 100429 (VDRC). ***Rdl^1^***: *Rdl[1]/TM3, Sb[1]*. ***Rdl^CB2^***: *Rdl[CB2]/TM6B, Tb[1]*. ***Rdl^CB-2L^***: *Rdl[CB-2L]/TM6B, Tb[1].* ***Rdl^MD-RR^***:*Rdl[MD-RR].* **Deficiency Stocks for viability screen:** Most of the deficiency stocks were obtained from Bloomington *Drosophila* Stock Centre (BDSC), and some DrosDel strains were a generous gift from Dr Guisy Pennetta, University of Edinburgh.

### Determining the lethal stage of *Adar 3/4S* OE

To collect eggs, ∼50 female and 30 young male *Adar 3/4S* OE flies were placed into an egg-laying chamber on a yeasted grape juice plate. After 6 h, the parent flies were removed, and the number of eggs counted. The number of second instar larvae was counted after another 60 h, and the number of pupae was counted on day 7 after the egg laying. The number of eclosed adults was counted until day 13.

### Viability screen

*w^1118^; Actin 5c-GAL4 /SM5 CyO; UAS-Adar 3/4S, Tub-GAL80^ts^,* virgin female flies were collected at 18°C, crossed with deficiency-bearing male flies at 27°C, and the number of progeny of each genotype was counted. Twelve to sixteen virgin female flies were crossed with 5–8 male flies in each standard vial. The parent flies were transferred three times at 2-day intervals, and their progeny were collected only up to 12 days after mating to avoid counting any second-generation progeny. To estimate the viability, the number of viable *Adar 3/4S* overexpressing progeny flies obtained in the heterozygous deficiency background was divided by the number of sibling heterozygous deficiency flies that had the *SM5 Cy* second chromosome rather than the *Actin 5c-GAL4* driver second chromosome and did not overexpress *Adar 3/4S*. Statistical significance of rescue was calculated with the binomial test to compare the viability of the surviving adult flies with the expected un-rescued viability of 0%. Statistical significance was further corrected using False Discovery Rate correction (FDR, Bonferroni, otherwise stated).

### qRT PCR

cDNA made from 500 ng of RNA with oligo-dT primers was used for quantification, with minus RT control and water-only negative controls. qRT PCR was performed with SYBER GREEN master mix, with either a BioRad (C1000™ Thermal Cycler) instrument, or a Light Cycler® 480 (Roche). All primers were tested for correlation factor and efficiency. All qRT PCR results were normalized to *Gapdh* level and to additional standards. *P*-values were calculated with the unpaired two-tailed Student’s *t*-test. For each comparison, the cDNAs used were made at the same time with the same amount of RNA. The PCR primers used for PCR are *Adar* forward: TGGACCTTCAGTGCAATCA, reverse: CCTCACCGGACTCGATTT.

### Editing assay

cDNA made from total RNA was amplified with specific primers for the regions of interest in the transcript. The PCR product was either directly sequenced or cloned into pGEM-T Easy and individual clones sequenced. Two methods of quantitation for editing efficiency were used. The first method involved sequencing of total RT-PCR product pools and measuring relative peak heights at the edited site. Editing levels of PCR products were measured by comparing the heights of the Adenosine and Guanosine peaks at the same position (Editing percentage = the height of Guanosine peak/the height of both Guanosine + Adenosine peaks). Three PCR sequences were used to estimate an average editing level. For the second method of editing quantification, 60–100 colonies were picked and each clone was sequenced with both T7 and SP6 primers. *P*-values were calculated using the Mann–Whitney U test.

### Sequencing primers

***sloND_F:*** GCGGGCATTATACATCTGCT. ***sloND_R:*** CGAGCAGAAAGAACACGAGA. 

s***loSG_UTR_F1:*** GCCAATGTGCCCATGATAAC. ***sloSG_UTR_R1:*** TTGGGATGGACAAAATACACC. ***sloSG_R2:*** ATCAGCGTTAAGGCGTTTTG***.sloUTR_F2:*** CGTACATTTGAACGATGGAGAA***.cg33205F1:*** TGACCACTAACGACGCCATA***.cg33205R1:*** CGCATCGTTTCCATTTCATT***.cg33205R2:*** CGCATCGTTTCCATTTCAT***.Rdl561F:*** TAAACATATCCGCTATTCTCGACTCC***.Rdl960R:*** GGCGATCCATGGGGAAATATTGTAG***.Rdl961F:*** AGCTGTGCCACATTGAAATCGAAAGC***. Rdl1680R:*** TGTGGGCGTGGTGTCCATGCCCGTG. 

s***yt1381F:*** CGTTGAAGGAGAGGGCGGACAG***.syt1860R:*** CCTTACTTCATGTTCTTCAGGATCTC***.Ca_alpha1D_F:*** CGTTGATGGAGAGGGCGGACAG***.Ca_alpha1D_R:*** GCAATGTGAAACAGTGGCACCATGGC.

### Drug administration

Clonazepam (SIGMA-ALDRICH, C1277) stock solution (63.2 mM in acetone) was diluted in distilled water to obtain a series of concentrations from 1.58 to 15.8 mM. Valproic acid (SIGMA-ALDRICH, P4543) 2-mM stock solution was made in water prior to use, and further diluted to 0.1–1 mM in water. 150 µl of the final drug dilution was added on the food surface in a standard food vial, and the food was subsequently mashed. Mated flies were placed in the drug-containing vial once most of the liquid had evaporated. Flies were transferred to fresh drug-containing food every 2 days and the number of eclosed male progeny was counted over 15 days.

### Climbing analysis

A column of 1.5 cm diameter and 20 cm height, cut from a 25-ml plastic pipette, was used for the climbing assay. The height of the column was divided into 120 equally spaced lines. For each test, one 2-day-old fly was placed into the column. The highest line the fly reached in 1 min was recorded. For each genotype, 6-10 individual flies were tested three times to acquire an average score. A two-tailed Welch *t*-test was performed to calculate the *P*-value compared with the *Adar ^5G1^* null fly group. The climbing assay was performed at room temperature, at the same time each day.

### *In vivo* extracellular recordings from larval motor neurons

Intact CNS from wandering third-instar larvae was dissected under external saline, leaving imaginal discs and peripheral nerves attached (30). An isolated CNS was positioned on top of a cured Sylgard-coated coverslip (SYLGARD® 184 Silicone elastomer kit). The CNS was placed with its dorsal surface uppermost and immobilized with tissue glue (Vet Bond, WPI) applied to stretched peripheral nerves.

To access motor neuron somata, a patch pipette (∼10 µm opening) filled with 1 mg/100 µl protease (type XIV from *Streptomyces griseus*, 1 mg/ml, Sigma), diluted in external saline, was used to make a small hole in the glial sheath on or near the midline of the CNS (31). Once neuron somata were free from attached glia, the enzyme pipette was removed, and a patch pipette filled with external saline was used for extracellular current recording. Gentle suction was applied to achieve a loose-seal on the aCC soma (identified by GFP). Recordings were made for 5 min with an Axopatch 200B amplifier in voltage clamp mode (V_hold_ 0 mV, Molecular Devices). Recordings were digitised at 20 KHz with an Axodata 1332A A/D board and collected with pClamp software.

For each genotype, four to eight neuronal extracellular current records were made. The analysis used 3-min recording periods taken shortly after the second minute of recording data. Counting the number of events (action potentials) was performed automatically in Clampfit. A burst was defined as a minimum of four events within an interval of 25 ms. *P*-values between different groups were calculated with the Poisson exact test.

## RESULTS

### Overexpression of adult *Adar 3/4* S isoform is lethal in *Drosophila*

The lethality caused by overexpression of the adult *Adar 3/4 S391* isoform in larvae and pupae provides an opportunity to perform a genetic screen to identify interacting genes in which mutations prevent lethality. Lethality depends on the RNA editing activity of the expressed dADAR since expressing an *Adar 3/4 E329A* construct, which encodes a catalytically inactive protein, or expressing the ‘edited’ dADAR 3/4 S391G isoform do not cause lethality (29). Genes suppressing the lethality might be key targets of RNA editing, or they may encode proteins that increase dADAR editing activity. Suppressor genes may also encode proteins that alleviate the critical toxic physiological effect of dADAR overexpression by some other means.

**Figure 1.**
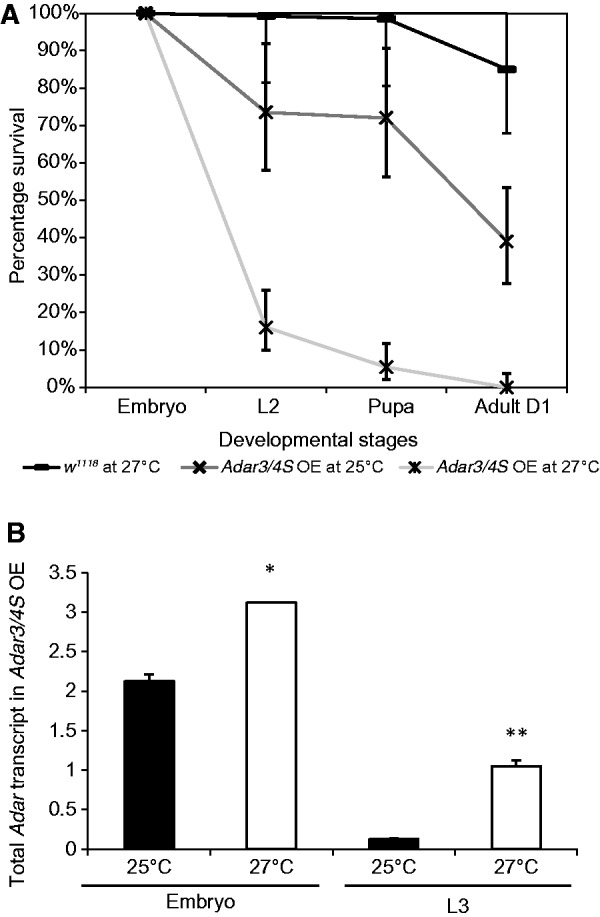
*Adar 3/4 S* overexpression causes lethality. (**A**) Survival of embryos, larvae and pupae of *Adar 3/4 S OE* at 25°C (*n* = 200) and at 27°C (*n* = 772) compared with *w^1118^* wild-type at 27°C (*n* = 400). Error bars represent Poisson 95% confidence intervals (**B**) *Adar* mRNA levels relative to *Gapdh* at 27°C versus 25°C in *Adar 3/4 S OE* whole embryos and L3 larvae. Three independent replicates were performed for qRT-PCR. The bars represent the mean value after normalizing to *Gapdh* expression levels. Error bars are SEM. Significance is indicated above the horizontal lines, *FDR *P* = 0.05, **FDR *P* = 1.841E-6 (two tailed Student’s *t*-test).

To perform this screen for genetic modifiers, the expression of the lethal dADAR 3/4 S protein was controlled by generating a strain that stably encodes both the *Actin 5C-GAL4* driver and the *UAS-Adar3/4 S* construct by addition of a *Tub-GAL80^ts^* construct (this strain will be referred to as *Adar 3/4S OE*). The GAL80^ts^ repressor protein is expressed constitutively in all cells from the *tubulin* promoter and binds to GAL4 to prevent transcription activation from the UAS (upstream activating sequence). At 25°C or lower, the temperature-sensitive GAL80^ts^ protein prevents expression of *Adar 3/4S* by binding to GAL4, thereby allowing adult flies to be obtained (32). At 27°C or at higher temperatures the GAL80^ts^ protein is no longer repressive, *Adar 3/4S* is expressed and no larvae or pupae survive to adulthood. Raising the temperature to 27°C has little effect on the viability of *w^1118^* wild-type flies (Figure 1A).

The lethality of *Adar 3/4S OE* occurs throughout larval and pupal stages (Figure 1A). RT-PCR analyses show that during embryonic stages at 27°C, the level of total *Adar* transcript in *Adar 3/4S OE* is approximately 1.5× higher on average than at 25°C whereas total *Adar* transcript is 7× higher at 27°C than at 25°C during the L3 larval stage (Figure 1B). RT-PCR primers are complementary to the *Adar* coding region and detect both endogenous *Adar* transcript and transcript expressed from the *Adar 3/4S* cDNA construct. *Adar* transcripts are normalized to *Gapdh*. Interestingly, *Adar 3/4S OE* flies raised at lower temperatures can survive at higher temperatures after they have eclosed from the pupae, which implies that the lethal effect of ADAR overexpression is limited to the earlier developmental stages when RNA editing levels are normally lower (data not shown). The viability of the adult flies facilitates the screen for genetic modifiers because it permits crosses with them to be performed at any temperature.

**Figure 2.**
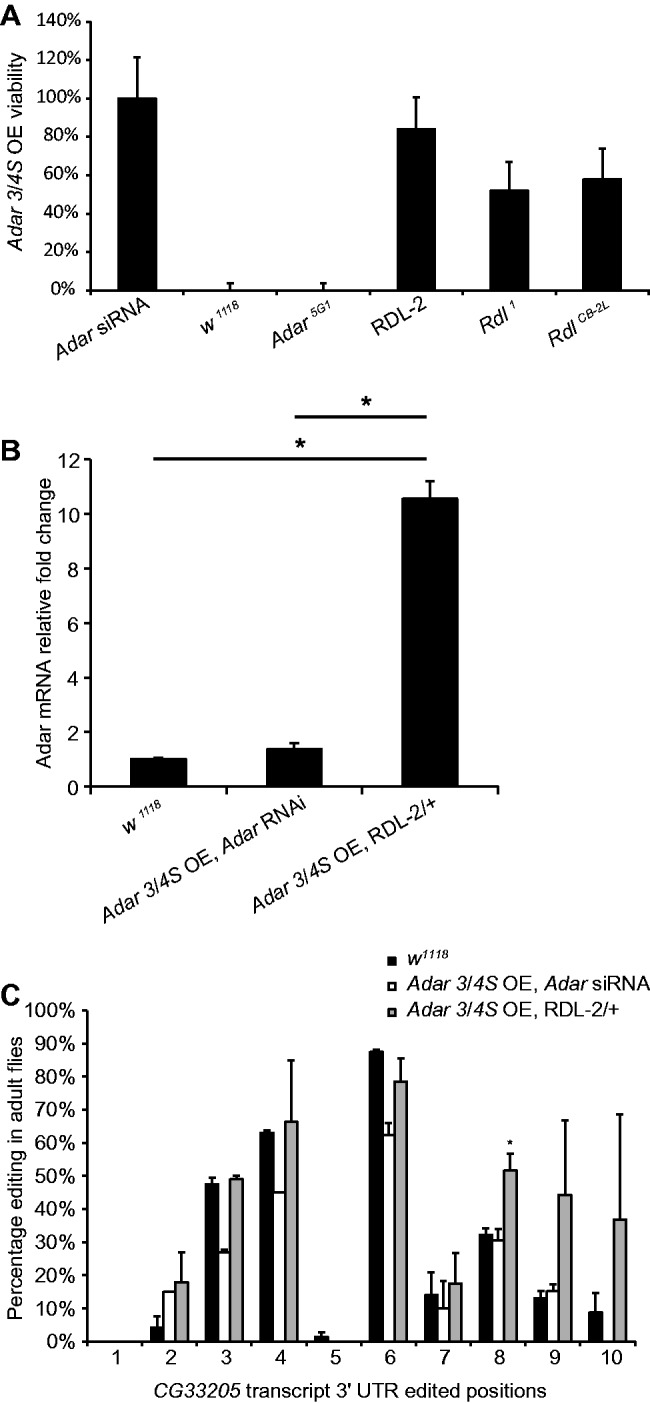
The Df(3L)RDL-2 deficiency rescues *Adar* overexpression lethality without preventing *Adar* overexpression. (**A**) The Df(3L)RDL-2 deficiency and *Rdl* mutants rescue lethality caused by *Adar 3/4S* overexpression. The genotype of the flies crossed with *Adar 3/4S OE* is displayed on the X axis. Error bars show 95% confidence limits (Poisson Exact Test). All the lethality-rescuing deficiencies were significantly different to the expected 0% viability for *Adar 3/4 S OE* flies (FDR *P* < 1.0e-312, Binomial Test). (**B**) Relative *Adar* mRNA level in *Adar* overexpression adult flies rescued by *Adar* RNAi or Df(3L)RDL-2 compared with that in wild-type *w^1118^*. The expression level of *Adar* was normalized to *Gapdh* before comparing between genotypes. Error bars are SEM. The significance of different comparisons is indicated above the horizontal lines,*P = 0.039 (two-tailed Student’s t test). (**C**) Editing levels at 10 sites in the 3’UTR of *CG33205* in *Adar 3/4S OE* adult flies rescued by *Adar* RNAi or Df(3L)RDL-2. Error bars are SEM. *P = 0.034 (Welsh *t*-test).

A wild-type *Adar* gene copy is present on the X Chromosome in the *Adar 3/4S OE* strain but crossing in the *Adar^5G1^* deletion mutant that deletes the *Adar* gene entirely does not rescue lethality at 27°C of male *Adar^5G1^* deletion progeny overexpressing *Adar 3/4S* ubiquitously (Figure 2A). Therefore, lethality is not mediated through the endogenous *Adar* gene and does not depend on additional expression of the endogenous transcript nor on any feedback effect on expression of *Adar* isoforms from the endogenous locus. Overexpressing an *Adar* RNA hairpin (*Adar* siRNA) fully rescues the *Adar 3/4S OE* lethality, confirming that lethality is owing to excess *Adar* (Figure 2A).

### A genetic screen identifies chromosomal deficiencies that rescue lethality caused by *Adar 3/4S* overexpression

To identify genetic modifiers of *Drosophila Adar 3/4 S OE* lethality, we performed a forward genetic screen by crossing *Adar 3/4 S OE* adult virgin flies from stock maintained at 25°C with males of heterozygous deficiency lines and raising the progeny at non-permissive temperatures >25°C. When the *Adar3/4S OE* flies were crossed at 29°C to flies of 128 stocks bearing chromosomal deficiencies that cover approximately 70% of Chromosome 3 in large blocks (33), no progeny overexpressing *Adar 3/4*S eclosed from pupae. When the crosses were repeated at 27°C, three heterozygous deficiencies on Chromosome 3L rescued the lethality (Supplementary Figure S1, Supplementary Table S1). We found three other weakly rescuing deficiencies on Chromosome 3R (Supplementary Table S1). A similar screen of deficiencies on Chromosomes 1 and 2 did not identify any further rescuing deficiencies. We focused our research on the Df(3L)RDL-2 deficiency because it rescued the lethality of *Adar 3/4S* OE most efficiently, to 90% of the level obtained with *Adar* RNAi (FDR *P* < 1.0E-312, Figure 2A). One lethality suppressor we identified on Chromsome 3R was the ubiquitin ligase *slmb* (*supernumerary limbs*); however, we did not study it further. Heterozygous mutation in *slmb* prevented *Adar* transcript overexpression. Reduced dosage of this ubiquitin ligase may affect the GAL4/UAS expression system, possibly by contributing to turnover of the GAL80^ts^ protein at nonpermissive temperatures (data not shown). We were unable to map the effects of some other rescuing deficiencies down to single genes, as testing rescue by overlapping deficiencies gave uninterpretable outcomes. This is a significant difficulty that frequently arises with deficiency screens and the reasons for such inconsistencies are not understood. The Df(3L)RDL-2 deficiency is a gamma ray-induced chromosomal deletion at the 66F5 region that deletes *Rdl (Resistant to dieldrin), nwk (nervous wreck), Tequilla* and *S(CypE^JP^)3.4* (34) (flybase: http://flybase.org/reports/FBab0024018.html).

The most interesting suppressors are those that allow continued overexpression of *Adar* while preventing the lethal effect. To determine whether the Df(3L)RDL-2 deficiency rescues without preventing *Adar* overexpression, we compared *Adar* transcript levels in rescued *Adar3/4S OE* flies heterozygous for the Df(3L)RDL-2 deficiency with *Adar* transcript levels in wild-type and in *Adar3/4S OE* flies rescued by RNAi against the overexpressed *Adar*. The Df(3L)RDL-2 deficiency-rescued flies had highly elevated expression of *Adar* transcript, with about 10-fold more *Adar* transcript in whole flies compared with *w^1118^* wild-type flies or *Adar3/4S OE* flies rescued by RNAi against *Adar* (Figure 2B). Therefore, it appears that rescue by Df(3L)RDL-2 is not caused by reduced expression of the *Adar* transcript, but is likely to involve mitigation of the effects of *Adar* overexpression. Because of *Adar3/4S* OE lethality, we could not compare the expression level of *Adar* transcript in the rescued adult flies with the level in *Adar3/4S* OE adult flies obtained through the same rearing protocol at 27°C.

Increased editing at sites in larval transcripts is a possible cause of the lethality observed on overexpression of highly enzymatically active adult ADAR 3/4 S isoform during embryonic and larval stages. This inappropriate editing could lead to expression of adult protein isoforms, which would mediate the lethal effect of *Adar* overexpression. We previously reported increased editing of the *Ca-α1D* transcript encoding a voltage-gated calcium channel in muscle and CNS in *Adar 3/4S*-overexpressing larvae (35).

Increased editing may still occur in the Df(3L)RDL-2-rescued adult flies, as they express elevated levels of *Ada*r. To determine whether *Adar 3/4S* OE flies rescued by the Df(3L)RDL-2 deficiency have above-normal RNA editing activity in some transcripts, we examined editing in the *CG33205* transcript from rescued adult flies. We chose this transcript because it has 10 edited sites with low or moderate editing levels close together in the 3’UTR. Its editing profile gives us an advantage in evaluating the editing level using a single pair of primers only, as most individual RNA editing sites require a separate set of RT PCR products for each site. The *CG33205* transcript is of no other particular interest except that it is widely expressed and might be edited even outside the nervous system in *Adar 3/4S* OE. One site (Position 8) showed a significant increase (1.65 fold, *P*≤ 0.034) in the editing level in *Adar 3/4S* OE; Df(3L)RDL-2 flies (Figure 2C), indicating that the overexpressed *Adar 3/4S OE* does lead to increased editing activity. We do not expect to see above-normal RNA editing at all editing sites, as previously shown (21,36). Preventing *Adar* overexpression by RNAi against *Adar* prevents increased editing in the *CG33205* transcript. Indeed *Adar 3/4S* OE; *Adar* RNAi shows a trend for reduced editing at some sites below that seen in *w^1118^* wild-type flies, which may reflect reduction in isoforms produced from the endogenous *Adar* locus. These findings show that heterozygous Df(3L)RDL-2 rescues the *Adar 3/4S* OE lethality even though *Adar* is still highly overexpressed and still maintains above-normal editing activity.

### Reduced *Rdl (Resistant to dieldrin),* gene dosage or *Rdl* mutants rescue *Adar 3/4S* overexpression lethality

Among the candidate genes in the Df(3L)RDL-2 deficiency, only mutations in *Rdl* were able to rescue the lethality of *Adar 3/4S* OE when tested. Heterozygosity for the *Rdl* null allele *Rdl^1^*, a large intragenic inversion covering multiple exons in *Rdl* (37), rescues *Adar 3/4S OE* viability to 51.9% (*n* = 94; Figure 2A), supporting our conclusion that the effect of the Df(3L)RDL-2 deficiency is due to reduced expression of the *Rdl* gene. *Rdl^1^* is a gamma ray-induced inversion with both breakpoints within the *Rdl* transcript. *Rdl^1^* homozygotes do not produce any *Rdl* transcript, which results in late embryonic stage lethality (37). Two *Rdl* RNAi lines were also tested at 27°C, but we could not determine whether they rescue the lethality of *Adar 3/4S* OE. *Rdl* is an essential gene and knocking down *Rdl* ubiquitously from embryonic stages by combining the *Actin 5C-GAL4* driver with these *Rdl* RNAi constructs in an *Adar* wild-type background is also lethal.

The encoded RDL protein (also known as GABA_A_ receptor subunit beta) is the pore-forming subunit of the ligand-gated inhibitory GABA_A_ receptor which is a major class of inhibitory receptor in the fly. RDL mediates fast synaptic inhibition through GABA_A_ receptors and shares 30–38% identity with vertebrate GABA_A_ receptor beta subunits (38). RDL can form functional GABA_A_ receptors as a homomer when expressed in *Xenopus laevis* oocytes (38,39). Although the physiological compositions of the insect GABA_A_ receptors are not clear, the homo-oligomeric GABA_A_ receptor formed by RDL subunits expressed in *Xenopus* closely resembles native insect neuronal GABA_A_ receptor in pharmacological sensitivities (40,41). The *Rdl* transcript is also alternatively spliced and alternate splice forms in combination with RNA editing profoundly influence GABA-mediated inhibition (25).

To confirm that mutations affecting RDL channel activity rescued *Adar* overexpression lethality, the *Adar 3/4 S OE* flies were crossed with three additional *Rdl* mutant lines-*Rdl^CB-2L^, Rdl^MD-RR^* and *Rdl^CB^*. All three are ethyl methanesulfonate (EMS)-induced *Rdl* mutations causing amino acid replacements; the *Rdl^CB-2L^* M267I mutation is on the extracellular part of the channel whereas *Rdl^CB-2^* and *Rdl^MD-RR^* change single amino acids at several sites in the intracellular part of the channel (http://flybase.org/reports/FBgn0004244.html) (Supplementary Figure S2). *Rdl^CB-2L^/+* rescued lethality by 57.9% (*n* = 55, FDR *P* < 1.0E-312; Figure 2A) but neither of the other alleles did. A limited number of *Rdl* mutations are available, but the data are consistent with the hypothesis that RDL channel function is the basis for rescue.

### No significant increase in editing is observed in the *Rdl* transcript in *Adar 3/4S* overexpressing or Df(3L)RDL-2-rescued animals

**Figure 3.**
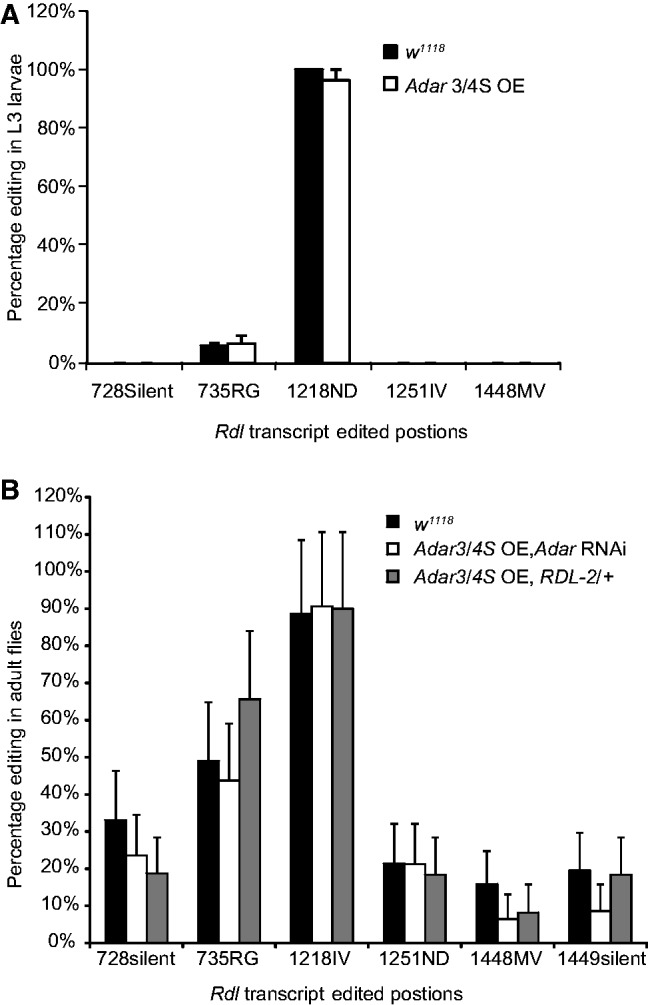
No significant increase in editing in the *Rdl* transcript in *Adar 3/4S* overexpressing or Df(3L)RDL-2-rescued animals. (**A**) Editing levels at sites in the *Rdl* mRNA in L3 larvae of *Adar 3/4S OE* or *w^1118^*. The horizontal axis shows the edited positions. The editing sites are named for the codon numbers and the resulting amino acid changes. Error bars are SEM. (**B**) Editing levels at sites in the *Rdl* mRNA in rescued *Adar 3/4S OE* or *w^1118^* flies. Error bars are SEM.

We investigated editing levels at sites in *Rdl* transcripts in *Adar3/4S OE* L3 larvae at 27°C. Among the five known edited sites, only the 735RG site showed a trend of increased editing (6.5–10.5%; Figure 3A). The 1218IV site, which is 100% edited in wild-type, is edited about 100% in *Adar3/4S OE*, and the other sites that are not edited in wild-type are also not edited in *Adar3/4S OE* (Figure 3A). In rescued adult *Adar 3/4S OE*; Df(3L)RDL-2 and *Adar 3/4S OE*; *Adar* RNAi flies, editing levels at sites in the *Rdl* transcript were not significantly different from *w^1118^* (Figure 3B)*.* Thus, we do not have evidence showing that altered editing of the *Rdl* transcript underlies *Adar 3/4S OE* lethality, although editing may influence expression of RDL channels.

### Increasing or decreasing GABA signalling causes reciprocal effects on phenotypes of *Adar^5G1^* null mutant and *Adar3/4S OE* flies

**Figure 4.**
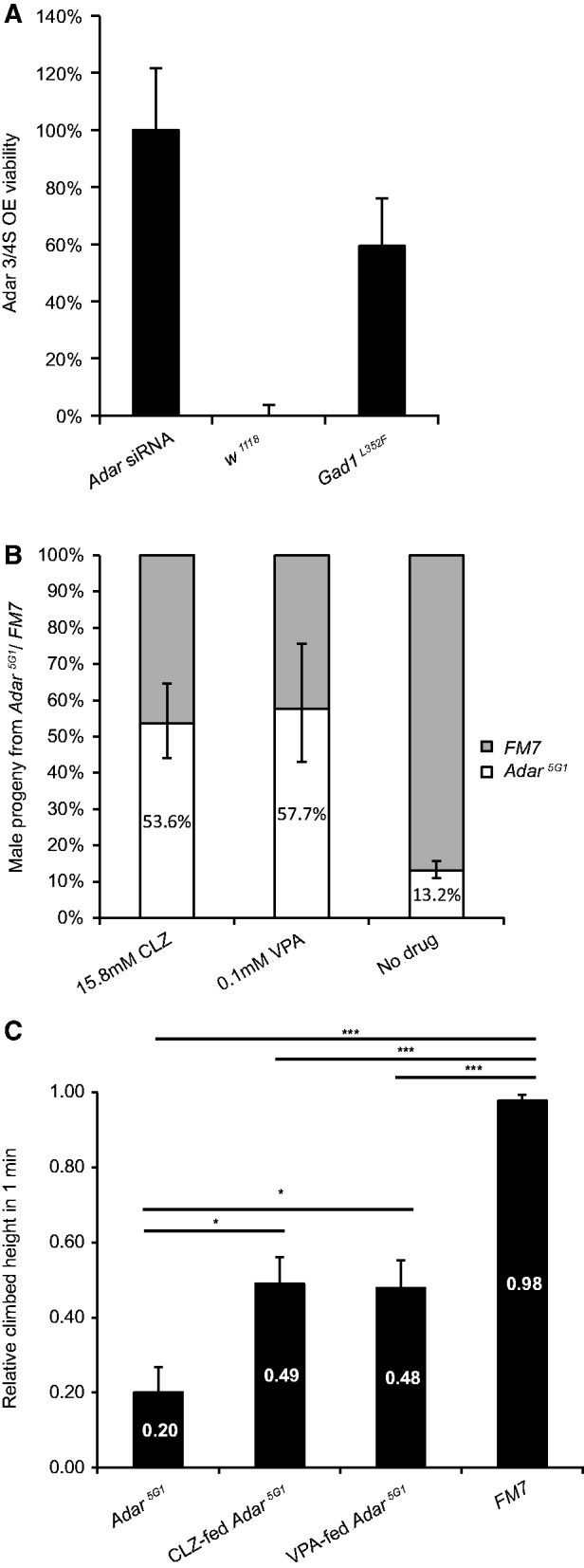
Reciprocal effects of modulating GABA signal strength on *Adar^5G1^* mutant or *Adar 3/4S* overexpression phenotypes. (**A**) Heterozygous *Gad1^L352F^* mutant rescues the *Adar 3/4S OE* lethality. The X axis show the genotypes of the flies crossed with *Adar3/4S OE* flies. The Y axis shows the viability of the F1 progeny that overexpress *Adar 3/4S*, compared with the rescue by *Adar* siRNA. Error bars show 95% confidence limits (Poisson exact test). All the lethality-rescuing deficiencies were significantly different to the expected 0% *Adar 3/4S OE* viability (FDR *P* < 1.0E-312, Binomial Test). (**B**) Increased relative viability of *Adar^5G1^* null flies compared with sibling FM7 flies among progeny of the *Adar^5G1^/FM7* stock raised on food with 15.8 mM clonazepam or 0.1 mM valproic acid. Error bars show 95% confidence limits (Poisson exact test). (**C**) Improved climbing activity of 2-day-old *Adar^5G1^* null flies raised on 15.8 mM clonazepam or 0.1 mM valproic acid. *FDR *P* < 0.02, ***FDR *P* < 1.0E-4 (two-tailed Welch t test). The horizontal lines indicate the comparison being made in each case. For each group, 10–13 individual flies were tested. Error bars: SEM.

To test whether the rescue of *Adar 3/4S OE* lethality by *Rdl* mutants was due to a reduction in GABA signalling, we examined the effect of decreasing GABAergic input on *Adar 3/4S OE* lethality. *Gad1^L352F^* is a strong hypomorphic mutant allele of *Gad1* (*Glutamic acid decarboxylase 1*), which encodes the enzyme that synthesizes GABA. The heterozygous *Gad1^L325F^* mutant has approximately 50% of the enzymatic activity of wild-type *Gad1*, and this is predicted to lead to reduced GABA inhibitory signalling (42). Crossing *Adar 3/4S OE* to *Gad1^L325F^* rescued lethality to 59.5% (*n* = 107, FDR *P* < 1.0E-312; Figure 4A), demonstrating that the decrease in GABA inhibitory signalling can be the mechanism underlying rescue by reducing *Rdl* expression.

Because reduction in *Rdl* expression or GAD1 activity rescued the lethality caused by *Adar 3/4S* overexpression, we sought a reciprocal rescue of *Adar^5G1^* null mutant phenotypes by chemically enhancing GABA signalling. Clonazepam (CLZ), is a benzodiazepine drug that binds to the benzodiazepine site of the GABA receptors, increasing the effect of GABA binding on neurons (43). CLZ has also been shown to weakly activate GABA_A_ receptors in insects (44). We also fed *Adar^5G1^* with a histone deacetylase inhibitor, Valproic Acid (VPA). VPA has a wide spectrum of activities including enhancing GABA neurotransmission by inhibiting GABA transaminase, and this function is conserved in lower animals including *Drosophila* (45,46). *Adar^5G1^* null male flies have significantly reduced viability compared with *FM7* male balancer siblings among progeny of *Adar^5G1^/FM7* females (Figure 4B). The FM7 male siblings have a wild-type *Adar* gene on a balancer first chromosome with multiple chromosomal inversions marked with a dominant *Bar* eye marker. *Adar^5G1^*/*FM7* flies were reared on either 1.58–15.8 mM clonazepam (CLZ) or 0.1–20 mM VPA-containing food to ensure that parent flies were exposed to the drug. These flies were then allowed to lay eggs on drug-containing food. Both CLZ (15.8 mM × 150 µl) and VPA (0.1 mM × 150 µl) increased the viability of *Adar^5G1^* null flies relative to *FM7* siblings among the progeny in the next generation (Figure 4B).

*Adar^5G1^* adult flies display locomotion defects and have reduced ability to climb up in a narrow column (Figure 4C). A fly in a narrow column climbs up to the top naturally, a response known as negative geotaxis (47). Most wild-type flies climb to the top in 20 s after being tapped to the bottom of the column but *Adar^5G1^* flies climb up to approximately 20% of the height of the column in 1 min (Figure 4C). *Adar^5G1^* null flies grown on the food containing high concentration (15.8 mM) CLZ or low dosage (0.1 mM) VPA showed statistically significant improvement in climbing ability by day 2 (FDR *P* < 0.02), although they were still defective compared with wild-type flies (FDR *P* < 1.0E-4; Figure 4C). Therefore, enhanced GABA signalling does ameliorate mutant phenotypes in *Adar^5G1^* flies, consistent with the hypothesis that these flies have increased excitability due to loss of RNA editing in neuronal transcripts.

### *Adar^5G1^* null mutant and dADAR-overexpressing larvae display reciprocal abnormalities in aCC motor neuron excitability

A possible explanation for the rescue of the *Adar 3/4 S OE* lethality by *Rdl* mutants is that lethality arises from suppressed neuronal excitability. We hypothesized that lethality is rescued by increased excitability due to an overall reduction in fast inhibitory GABA signalling through the RDL channel. If this hypothesis is correct, the *Adar 3/4S OE* flies would have lower neuronal activity while *Adar* null flies would have higher neuronal firing activity.

**Figure 5.**
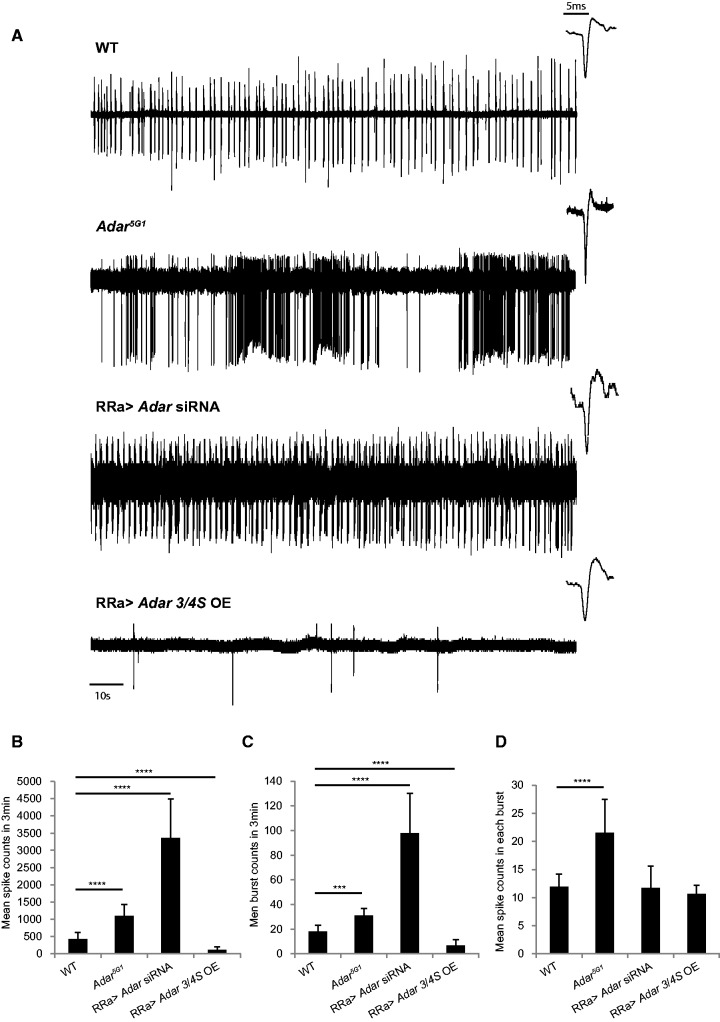
Reciprocal effects of decreased or increased dADAR expression on motor neuron excitability in *Adar^5G1^* mutant or *Adar 3/4S*-overexpressing larvae. (**A**) Examples of single aCC neuron activity recordings from wild-type, *Adar^5G1^*, *RRa>Adar siRNA* and *RRa>Adar 3/4S OE* larvae viewed over 3-min time scales. On the right top corner of each spike train is a 5 ms zoomed-in view showing true action potential spikes in the spike train. (**B–D**) Quantification of firing activities. The bars represent the averages of 4–8 recordings of different aCC cells. Error bars are SEM. ***FDR *P* < 1E-6, ****FDR *P* < 1E-50 (Poisson exact test). Genotypes are **WT:** *UAS-GFP; RRa- GAL4*. ***RRa>Adar 3/4S OE*:** *UAS-GFP/+; RRa-GAL4/UAS-Adar 3/4S*. ***Adar^5G1^*:** *Adar^5G1^; UAS-GFP/+; RRa-GAL4/+*. ***RRa>Adar siRNA*:** *UAS-GFP/+; RRa-GAL4/UAS-Adar siRNA*.

If phenotypes of *Adar* mutants or *Adar* overexpressors are due to aberrant excitability that can be corrected by manipulating GABA receptors then aberrant excitability is likely to occur in many different types of neurons that express *Rdl*. The larval motor neuron preparation gives access to a well-studied neuron in which we can measure the effects of increased or decreased *Adar* expression on neuronal excitability. The aCC motor neurons in the larval ventral ganglion project to dorsal muscles, receive GABA input, express *Rdl* (37) and can be identified when UAS-GFP is expressed specifically in these neurons under the control of an *RRa-GAL4* driver. We recorded endogenous action potential firing in aCC motor neurons of L3 larvae using an *in vivo* extra-cellular recording procedure. When additional *Adar 3/4S* is expressed in the *Adar* wild-type in the aCC motor neurons specifically using the *RRa-GAL4* driver, the aCC motor neurons show significantly reduced firing activity (Figure 5A). Reciprocally, aCC motor neurons of *Adar^5G1^* null mutants were hyper-active (Figure 5A). aCC motor neurons with *RRa-GAL4-*driven *Adar* siRNA knockdown in an *Adar* wildtype background were also hyper-active (Figure 5A).

The firing frequency, represented by the number of firing events (Figure 5B), and the number of bursts in 3 min (Figure 5C) were significantly decreased in *Adar 3/4S OE* aCC motor neurons (FDR *P* < 1.0E-6), but increased in *Adar^5G1^* null or *Adar siRNA* aCC motor neurons (FDR *P* < 1.0E-7). All these phenotypes are more severe in the *Adar* siRNA aCC motor neurons than in the *Adar^5G1^* null larvae. Therefore, these are likely to be cell-autonomous effects of dADAR level. We reason that *Adar^5G1^* larval aCC motor neurons show less severe phenotypes than *Adar* siRNA aCC motor neurons because non–cell-autonomous effects differ depending on whether other projecting neurons or niche cells also have *Adar* expression. The mean number of the events in each burst, however, was significantly increased only in the *Adar^5G1^* null larval aCC motor neurons (FDR *P* ≤ 2.27E-5, Figure 5D), indicating that, unlike the other effects, the effect on burst length is not cell-autonomous but is a systemic physiological response of the neurons. These *in vivo* extracellular recordings of *Adar* mutant larval aCC motor neurons strongly support the genetic data, indicating that a major physiological role of dADAR is to control neuronal excitability.

## DISCUSSION

Ubiquitous expression of a highly active adult ADAR isoform at larval and pupal stages is lethal in *Drosophila*. Taking advantage of the *GAL4/UAS* system, we placed the lethal *Adar* overexpression under temperature-dependent control, constructing a *Drosophila* strain that can be reared at 25°C for the purpose of performing a genetic screen. When progeny from this line (termed *Adar 3/4S* OE) or from crosses of this line to wild-type are reared at 27°C, all *Adar 3/4S* OE progeny die during pre-adult stages. *Adar 3/4S* OE females were crossed to males carrying defined chromosomal deletions, and we identified heterozygous deletions that prevent *Adar 3/4S* OE- induced lethality. The *Rdl* gene is responsible for the effect of one strongly rescuing deletion. *Rdl* is an essential gene encoding a pore-forming subunit of the important inhibitory GABA_A_ receptors in *Drosophila*.

Among the three tested *Rdl* point mutants, only *Rdl^CB-2L^* rescued the lethality of the *Adar3/4S OE* flies. This mutant has a single amino acid (M267I) change in the *N*-terminal extracellular region of the receptor. This M267I site may affect binding of GABA to the receptor; no other phenotypes have been observed for this mutant except that the homozygous mutant M267I *Rdl^CB-2L^* is lethal. It is clear that GABA signalling corrects *Adar* phenotypes. Enhancing the GABA signal in *Adar^5G1^* null flies by feeding flies with CLZ or VPA improved survival and early-stage fitness. Compared with CLZ, which is specific for GABA receptors, VPA has a much wider spectrum of effects, including increased autophagy. Nevertheless, both drugs improved *Adar^5G1^* fly defects to similar degrees, indicating that additional effects of VPA do not further improve mutant fly viability and locomotion.

The firing frequency of single motor neurons is controlled cell autonomously by dADAR. *Adar^5G1^* larval aCC motor neurons have increased firing frequency and knocking down *Adar* in aCC motor neurons further increases firing frequency. Overexpressing dADAR 3/4S in aCC motor neurons reduces spontaneous firing activity. These cell autonomous effects are more likely to be mediated mainly through the editing status of ion channel receptors on motor neuron membranes than by changes in upstream inputs or in presynaptic interneurons. However, the defect in cell-autonomous firing frequency control is more significant in the neurons with targeted knockdown of *Adar* than in the neurons from the *Adar^5G1^* null mutant larva. The physiological feedback on motor neurons from other cells with which they interact is likely to be affected by whether those cells also have *Adar*. We demonstrate that dADAR dampens neuronal spontaneous firing activity cell autonomously in concert with the GABA_A_ fast inhibitory signal and suggest that this is the likely basis for lethality rescue by mutations in *Rdl.*

Tonic and burst firing pattern control is likely to be modified by dADAR non-cell autonomously. *Adar^5G1^* larval motor neurons show prolonged bursts whereas knockdown of *Adar* in motor neurons did not change the length of each burst or the pattern of tonic and burst firing. We recorded aberrant excitability in *Adar^5G1^* null and *Adar 3/4S* overexpressing larval motor neurons, but it is likely that many other types of neurons are similarly affected. We do not know where in the body the lethal effect of *Adar 3/4S* overexpression is caused although it is likely to be in GABA-responsive neurons that express *Rdl.*

We do not fully understand how changes in ADAR dosage cause changes in excitability. The lethal effect of *Adar 3/4 S* overexpression is dependent on the high RNA editing activity of this isoform, and the increased editing at sites in larval transcripts that we reported previously has also been observed at further sites in this study. However, the number of identified sites of increased editing remains surprisingly limited. Editing levels at many sites appear relatively unresponsive to changes in dADAR levels or isoforms. A recent report from Savva *et al.* (36) examined the editing site specificity of dADAR S and dADAR G proteins expressed from the chromosomal *Adar* locus and found that most sites are edited similarly by the two ADAR isoforms, with only a few sites preferring dADAR S. Purified ADAR proteins edit individual RNA substrates specifically *in vitro*, but other proteins interacting with RNA or with dADAR may have strong effects on editing levels at individual sites *in vivo*. The rescue by altered *Rdl* dosage raises the question of whether altered editing of *Rdl* drives the changes in neuronal excitability seen in *Adar* mutants or overexpressors. *Rdl* transcripts are edited at one site in larvae and at additional sites in adult flies, but alterations in the pattern of editing in the *Rdl* transcript itself do not appear to be central to *Adar 3/4S* OE lethality. Indeed, *Rdl* editing at most sites increases the EC50 values for homomeric RDL channel responses to GABA agonists. If increased editing also makes native RDL channels similarly less responsive to GABA then effects of *Rdl* editing seem unlikely to mediate the neuronal excitability changes observed (25). Therefore, aberrant editing of transcripts other than *Rdl* may underlie the effects of increased or decreased dADAR on neuronal excitability.

**Figure 6.**
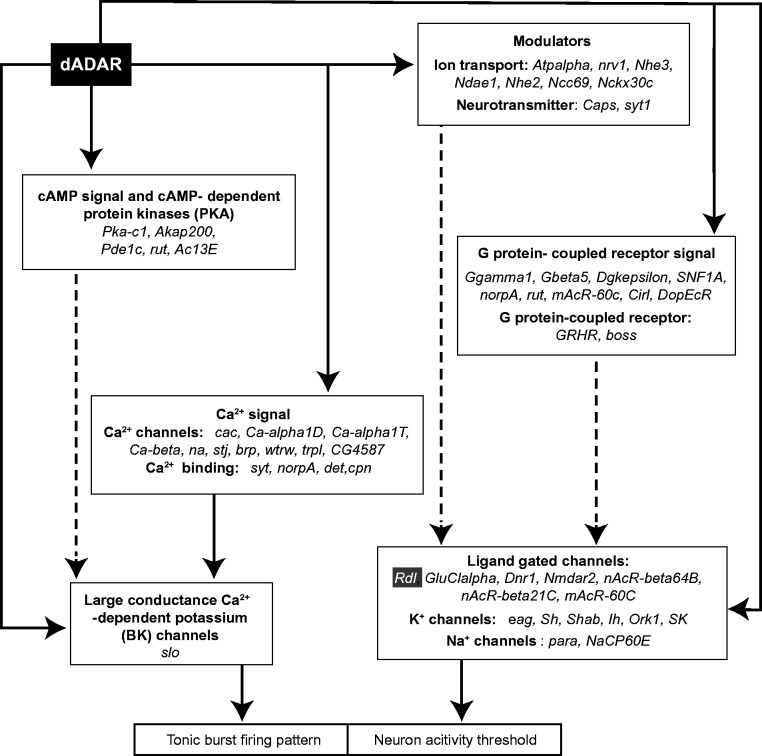
*Drosophila* CNS edited transcripts encode a range of proteins likely to underlie aberrant neuronal excitability in *Adar*-overexpressing or *Adar* null mutants. Solid lines indicate direct control and dashed lines show indirect effects. Each box represents a category of edited transcripts encoding proteins that either directly or indirectly influences neuronal excitability by effects on the action potential firing threshold or tonic or burst firing pattern control. Transcripts edited by dADAR encode ligand-gated channels, potassium channels and sodium channels that set the neuronal activity threshold. *Rdl*, encoding a GABA-gated GABA_A_ receptor subunit, is highlighted in the box at the right lower corner. Various transcripts encoding modulators, including ion transporters, neurotransmitters and G-protein coupled signalling proteins are also edited by dADAR. Functional changes in these proteins will also indirectly modulate the ion channels that determine the neuronal activity threshold. dADAR is also expected to modulate the neuron firing pattern. An edited *slo* transcript that encodes the Calcium-dependent large potassium (BK) channel is known to directly regulate the tonic or burst firing pattern of the neuron. Ca^2+^ directly regulates BK channel activity; therefore, all genes listed in the ‘Ca^2+^ signal’ box both influence BK channel activity and express transcripts edited by dADAR. In addition, some edited transcripts encode cAMP signalling and PKA family proteins that may indirectly control BK channel activities.

The reciprocity of the excitability effects of increased and decreased dADAR, however, raises the intriguing possibility that these effects arise from either too much or too little editing in the same set of transcripts. The effects of editing alterations on the function of each individual encoded protein may be mild (36), though cumulative effects may be significant (Figure 6). The frequency of neuron firing events is known to be controlled directly by large-conductance calcium and voltage-gated potassium channels (BK channels), and indirectly by calcium channels (48). The *slowpoke (slo)* transcript encoding the BK Ca^2^^+^-channel (Figure 6) has four editing sites*,* including two that result in amino acid changes in the extracellular vestibule and conducting pore of the channel (21,23,49). Many transcripts encoding synaptic release machinery are also edited by dADAR, including the *synaptotagmin* transcript encoding the Ca^2+^ sensor for synaptic vesicle fusion (23,50). The increase in the length of burst may be caused mainly by altered presynaptic Ca^2+^ release in the *Adar^5G1^* larvae, due to loss of editing in transcripts encoding neurotransmitter receptors and to altered abundance of synaptic release machinery as is observed in the NMJ of *Adar^5G1^* larvae (28). A full survey of editing events in *Adar 3/4S OE* will be required to search for key targets. The effects on excitability could also arise in more indirect ways through editing of microRNAs or repetitive RNA sequences leading to stress or immune responses (51–53).

We demonstrate for the first time that RNA editing by dADAR can fine-tune neuronal activities in concert with GABA fast inhibitory signalling. Many, though not all, of the effects of editing on ion channels in flies and vertebrates can be accommodated by the hypothesis that A-to-I RNA editing reduces neuronal excitability (15,27,54)*.* For instance, editing reduces calcium permeability of glutamate-gated AMPA channels, decreases serotonergic potency of 5HT_2C_ receptors and reduces the inactivation rate of the Kv1.1 channel (4,55,56). Therefore, RNA editing can create protein diversity though why it has evolved to specifically modulate neuronal excitability is a question that remains to be answered.

## SUPPLEMENTARY DATA

Supplementary Data are available at NAR Online.

## FUNDING

Medical Research Council (to M.O’C. and Centenary Award 42507 to X.L.); MND Scotland (to L. K.), (Prize Studentship Award 41945); Royal Society of Edinburgh Scottish Government Fellowship cofunded by the Medical Research Council and Marie Curie Actions (to I.O.). Funding for open access charge: Medical Research Council [Centenary Award 42507 to X.L.].

*Conflict of interest statement*. None declared.

## Supplementary Material

Supplementary Data
