# Supplementary material for: The ADAR RNA editing enzyme controls neuronal excitability in Drosophila melanogaster
Source: Nucleic Acids Res. 2013 Oct 16;42(2):1139–51. doi: 10.1093/nar/gkt909 (PMC3902911; doi:10.1093/nar/gkt909)
Supplement: Supplementary Data [file supp_42_2_1139__index.html]

The ADAR RNA editing enzyme controls neuronal excitability in Drosophila melanogaster — The ADAR RNA editing enzyme controls neuronal excitability in Drosophila melanogaster — Supplementary Data 

# The ADAR RNA editing enzyme controls neuronal excitability in *Drosophila melanogaster*

## Supplementary Data

files

**Files in this Data Supplement:**

- Supplementary Data - pdf file
- Supplementary Data - pdf file
- Supplementary Data - pdf file
