# Supplementary material for: The ADAR RNA editing enzyme controls neuronal excitability in Drosophila melanogaster
Source: Nucleic Acids Res. 2013 Oct 16;42(2):1139–51. doi: 10.1093/nar/gkt909 (PMC3902911; doi:10.1093/nar/gkt909)
Supplement: Supplementary Data [file supp_gkt909_nar-02014-a-2013-File008.pdf]

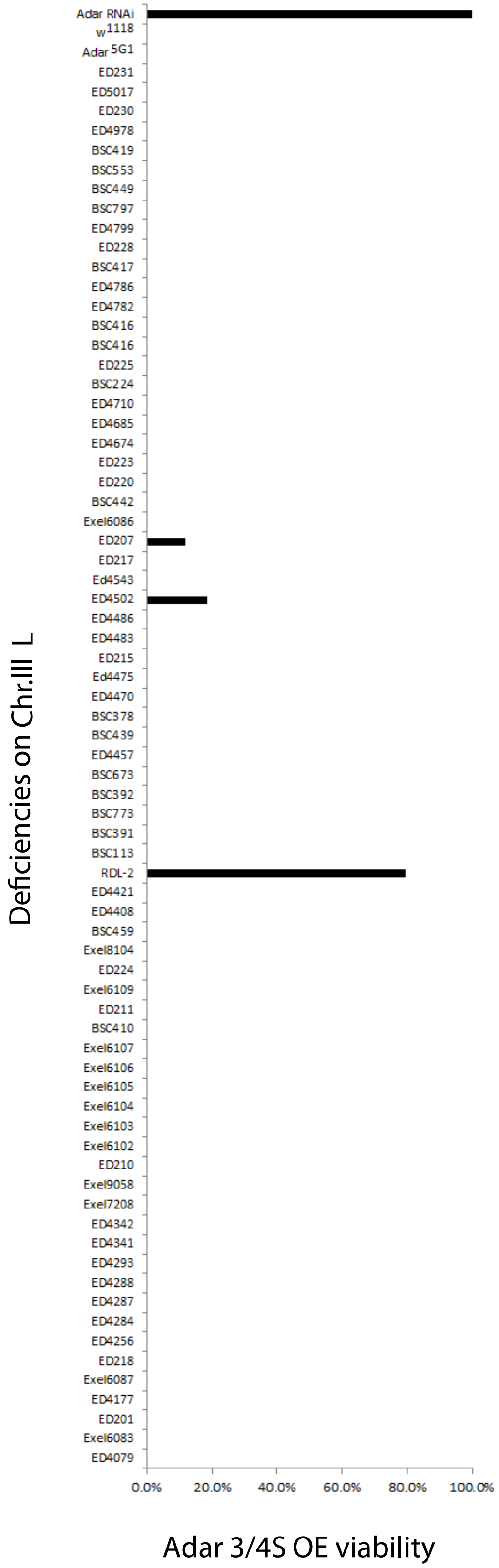

Supplementary Figure 1.

Deficiency screen on Chromosome III left arm for rescue of lethality caused by *Adar3/4S* overexpression. The y axis lists the deficiencies crossed with *Adar3/4S* OE and the horizontal bars show the viability of *Adar3/4S OE, Df* flies. The first three control columns on the y axis shows a full rescue by *Adar* RNAi and that no *Adar3/4S* OE progeny survive from crosses with w^{1118} wild type flies or from crosses with $Adar^{5G1}$ flies.
