# Supplementary material for: The ADAR RNA editing enzyme controls neuronal excitability in Drosophila melanogaster
Source: Nucleic Acids Res. 2013 Oct 16;42(2):1139–51. doi: 10.1093/nar/gkt909 (PMC3902911; doi:10.1093/nar/gkt909)
Supplement: Supplementary Data [file supp_gkt909_nar-02014-a-2013-File009.pdf]

Supplementary Table 1. Mapping the genes present in the deficiencies that rescue *Adar 3/4 S* OE lethality.

Deficiencies	Rdl-2	BSC508	ED5429	ED10639	ED5100	ED4502	ED207
**Including**	nwk, Rdl<br>S(CycEJP)<br>3.4<br>Tequila		Exel6153<br>Exel6264	Exel7329<br>Exel7330	ED5020<br>ED5095<br>ED5066<br>BSC174<br>Exel6141	BSC614	BSC431<br>BSC250
**Tested**	-	-	-	-	-	-	-
Negative:	nwk IR,<br>RdlIR	ED10820<br>ED10845<br>Rab11 IR	Exel6153<br>Exel6264<br>ED5454	Exel7329<br>ED10642<br>Pak3 IR	ED5020<br>ED5095<br>ED5066<br>BSC174	BSC614<br>ED4543	BSC289<br>ED4177
	+	+			+		
Positive:	Rdl^{1}<br>Rdl^{CB-2L}	slmb			Exel6141		
**Candidates**	*Rdl*	*slmb*	*trabid*<br>*dalmatian*<br>*hyd*	*taranis*<br>*Spineless*<br>*Oxidation reductase*<br>*etc.*	*Fip1*<br>CG31523<br>CG14651<br>ED5021<br>*Aux etc.*	*capricious*<br>*Acp70A*<br>*Rgl*<br>4 unknown genes	*Aplip1,*<br>*Mtacp1,*<br>*LysB~S,*<br>*etc.*<br>BSC431<br>BSC250
