# Supplementary material for: The ADAR RNA editing enzyme controls neuronal excitability in Drosophila melanogaster
Source: Nucleic Acids Res. 2013 Oct 16;42(2):1139–51. doi: 10.1093/nar/gkt909 (PMC3902911; doi:10.1093/nar/gkt909)
Supplement: Supplementary Data [file supp_gkt909_nar-02014-a-2013-File010.pdf]

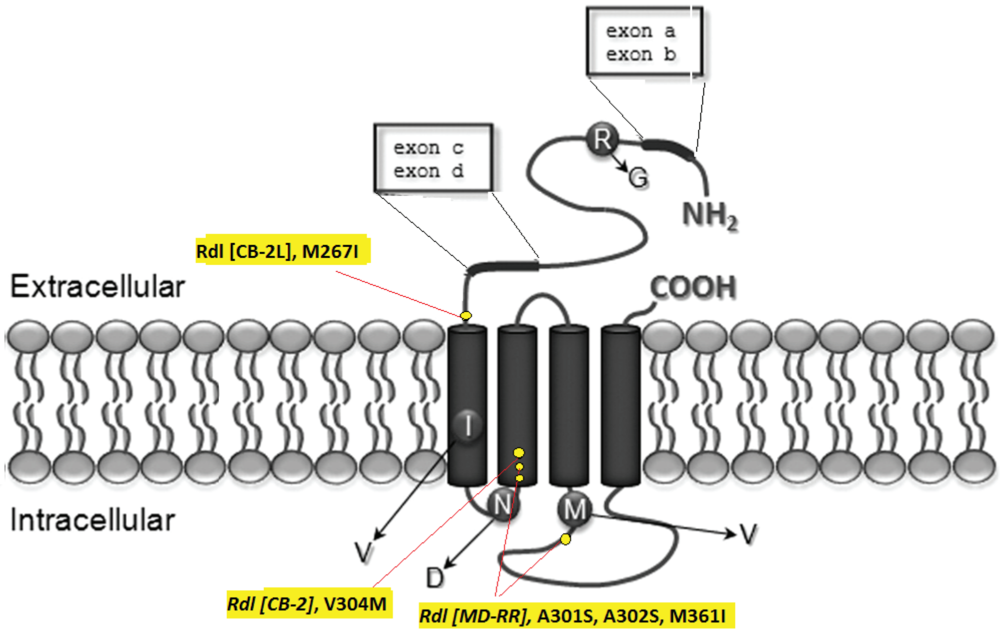

Supplementary Figure 2.

Schematic structure of RDL in the cell membrane and positions of alternative splicing sites, editing sites and point mutations. Two alternative splicing sites are in the extracellular region. Three *Rdl* mutants with their positions and amino acid changes are highlighted with yellow. Four editing sites are R122G, N294D, I283V and M360V. Figure adapted from Jones et al.,2009.
